# Trypsin in Cancer

**Published:** 1917-04

**Authors:** 


**Trypsin in Cancer.** M. W. McDuffie of N. Y., Med. Times, Feb., reports two cases treated by intravenous injections. One died of apoplexy from straining at stool and post mortem showed no evidence of remaining cancer, the other was clinically cured.

